# Immunostimulatory Gene Therapy Using Oncolytic Viruses as Vehicles

**DOI:** 10.3390/v7112899

**Published:** 2015-11-06

**Authors:** Angelica Loskog

**Affiliations:** 1 Department of Immunology, Genetics and Pathology, Science for Life Laboratory, Uppsala University, Rudbeck laboratory C11, Dag Hammarskjoldsvag 20, 75185 Uppsala, Sweden; angelica.loskog@igp.uu.se; Tel.: +46-735377161; 2 Lokon Pharma AB, Dag Hammarskoldsvag 40c, 75183 Uppsala, Sweden

**Keywords:** oncolytic, adenovirus, gene therapy, immunotherapy, tumor immunology

## Abstract

Immunostimulatory gene therapy has been developed during the past twenty years. The aim of immunostimulatory gene therapy is to tilt the suppressive tumor microenvironment to promote anti-tumor immunity. Hence, like a Trojan horse, the gene vehicle can carry warriors and weapons into enemy territory to combat the tumor from within. The most promising immune stimulators are those activating and sustaining Th1 responses, but even if potent effects were seen in preclinical models, many clinical trials failed to show objective responses in cancer patients. However, with new tools to control ongoing immunosuppression in cancer patients, immunostimulatory gene therapy is now emerging as an interesting option. In parallel, oncolytic viruses have been shown to be safe in patients. To prolong immune stimulation and to increase efficacy, these two fields are now merging and oncolytic viruses are armed with immunostimulatory transgenes. These novel agents are racing towards approval as established cancer immunotherapeutics.

## 1. Cancer Immunotherapy

**Figure 1:**
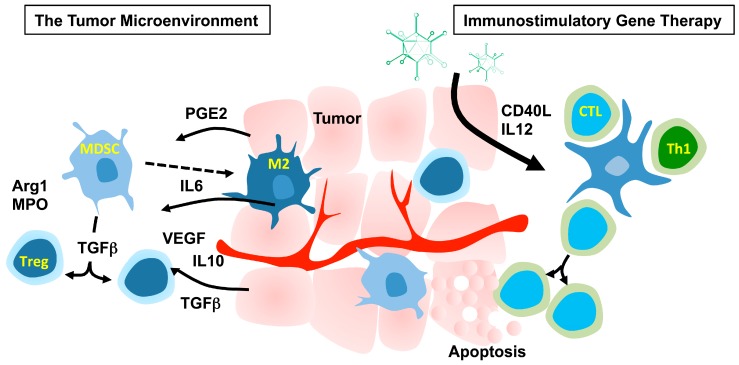
The tumor microenvironment consists of both tumor cells and other cell types such as immature myeloid-derived suppressor cells (MDSC), M2 macrophages and T regulatory cells (Treg). These cells act together to promote tumor progression and suppress anti-tumor immune reactions. The aim of immunostimulatory gene therapy is to shift the ongoing immunosuppression towards Th1 immunity by activating dendritic cells (DCs), T helper (Th)-1 cells and cytotoxic T lymphocytes (CTLs) to induce tumor-specific killing by lymphocytes. PGE2: prostaglandin E2; IL: interleukin; VEGF: vascular endothelial growth factor; TGFβ: transforming growth factor β; Arg1: arginase 1; MPO: myeloperoxidase; CD40L: CD40 ligand.

It has been known for decades that the immune system can recognize and eradicate malignant cells. Different types of immunotherapies have been evaluated in an attempt to boost the ongoing anti-tumor responses. Treatments like interferons, granulocyte macrophage-colony stimulating factor (GM-CSF) and bacillus Calmette-Guérin (BCG) were the first approved immunotherapeutics but with high toxicity or questionable response rates. Nevertheless, BCG has been approved for more than 30 years for superficial bladder cancer since it significantly prolongs the relapse free intervals in this indication [1]. Unfortunately, the tumor and its microenvironment counteract immune responses by inducing immunosuppressive cells like M2 macrophages, myeloid-derived suppressor cells (MDSCs) and T regulatory cells (Tregs) [2,3] (Figure 1). In the tumor milieu, activated cytotoxic T lymphocytes (CTLs) are rapidly suppressed by these cells and become anergic, a state of reversible unresponsiveness, or die. In experimental settings, many immunotherapies have been evaluated, but real success has been absent until the patients were preconditioned with chemotherapy and/or irradiation to remove some of the immunosuppressive cells prior to immunotherapy. For example, the treatment of malignant melanoma using autologous *ex vivo* expanded tumor-infiltrating T lymphocytes was not effective but when combined with preconditioning the objective response rates reached 72% [4]. Gene engineered chimeric antigen receptor (CAR) T lymphocytes have also shown spectacular results in B cell malignancy after the introduction of preconditioning strategies [5]. A different approach is to block the inhibitory signaling that would otherwise restrain CTLs by using checkpoint blockade antibodies targeting CTLA-4 (CTL-associated protein 4) or PD-1/PD-L1 (Programmed death-ligand 1 and its receptor) [6]. Naturally occurring anti-tumor T lymphocytes previously controlled by immune evasion strategies are then released from restraint and can recognize and kill tumor cells. Checkpoint blockade antibody treatment is now approved for many cancers [6,7] and the next step is to combine this “release of the break” treatment with activating immunotherapies to reach long-term anti-tumor immunity and increase the complete response rates in the patients. Hence, the essence of cancer immunotherapy is to break tumor tolerance (e.g., break anergy) and to revert the ongoing suppressive responses to instead activate anti-tumor immunity. Novel concepts to treat cancer by stimulating the immune system are currently being investigated. One of these concepts is immunostimulatory gene therapy utilizing viruses as gene delivery vehicles [8].

## 2. Immunostimulatory Gene Therapy

Immunostimulatory gene therapy aims to transfer genes coding for immunostimulatory proteins into the tumor area to induce tumor immunity. The first studies using immunostimulatory gene therapy in experimental models were published in the late nineties with promising results [9,10,11,12,13,14]. For example, the murine gene for CD40 ligand (CD40L) was introduced into murine neuroblastoma tumor cells using a retrovirus as a gene delivery vehicle. CD40L is one of the most potent stimulators of dendritic cells (DCs) and drives the formation of T helper 1 (Th1) type of immune responses with subsequent activation of CTLs. The expression of CD40L by the tumor cells made them highly immunogenic and significantly increased the survival of the mice after injection of modified tumor cells compared to the injection of wild type tumor cells. Not all cells needed to express CD40L to increase the survival. If less than 1.5% of the tumor cells expressed CD40L, it was still sufficient to induce a tumor immunity that was dependent on CD8+ T lymphocytes [9]. Similar findings were noted for interleukin (IL)-12 and other cytokines as well upon gene transfer to murine tumor cells [10,11,12,13,14]. The concept was also valid for gene engineered fibroblasts expressing immunostimulatory genes and was used to induce anti-tumor immune responses [15]. Hence, injection of gene therapy vehicles into the tumor microenvironment to express immunostimulatory proteins can elicit anti-tumor immunity even if not all tumor cells are targeted and even if other cell types in the milieu express the stimulators. The target of immunostimulatory gene therapy is the cells of the immune system such as DCs that have engulfed tumor debris or tumor-specific T cells. These cells travel to lymph nodes and adjacent metastases to elicit immune responses against the tumor cells. Hence, local administration of the gene vehicles into the tumor milieu will lead to activation of systemic anti-tumor immunity. This was in sharp contrast to the previous cancer gene therapies that focused on gene corrective treatment [16]. Thus, all tumor cells needed to be targeted by the gene vehicle to eradicate the tumor.

### 2.1. The Tumor Microenvironment

To understand which immune stimulators should be used for immunostimulatory gene therapy, the microenvironment within the tumor needs to be considered. The tumor cells and the tumor stroma can produce factors such as transforming growth factor (TGF)-β, IL1, IL6β, GM-CSF, tumor necrosis factor (TNF)-α, vascular endothelial growth factor (VEGF), chemokine (C-C motif) ligand 2 (CCL2), chemokine (C-X-C motif) ligand (CXCL)12 and CXCL15 [17]. These factors disturb the normal myeloid cell development in the bone marrow and immature myeloid cells are attracted to the tumor environment. These immature myeloid cells are collectively called MDSCs and are immunosuppressive since they produce inhibitory cytokines and growth factors such as IL10, TGFβ, Prostaglandin E2 (PGE_2_), Arginase I and myeloperoxidase (MPO) that are involved in both preventing DC maturation as well as exerting direct suppressive activity on lymphocytes. Many tumors are also infiltrated with mature myeloid cells such as M2 macrophages since monocytes entering the tumor adapt to the environment consisting of IL4, IL13, IL10, and TGFβ by differentiation into M2 macrophages secreting even more TGFβ, IL6, IL10, VEGF but also other factors such as IL17, IL23, fibroblast growth factor 2 (FGF2), a number of chemokines and matrix metalloproteases (MMPs) [18]. VEGF, FGF, IL17, IL23 and TGFβ, contribute to the induction of vascular endothelial cell proliferation while MMPs induce sprouting and migration of endothelial cells into the tumor, which lead to formation and maturation of new vessels. Thus, M2 macrophages are key players in angiogenesis and are necessary to provide the growing tumor with an efficient delivery of oxygen and nutrients. Furthermore, M2 macrophages express PD-L1 that can efficiently inhibit activated PD-1+ T cells [19]. The milieu created by the tumor and the myeloid cells differentiates naive CD4 T cells into FoxP3+ T regulatory cells that are also producers of IL10 and TGFβ. Hence, the tumor microenvironment is hostile to anti-tumor immune reactions. Nevertheless, despite the suppressive environment, the tumor is also alerting the immune system of ongoing danger due to hypoxia, tissue disruption and cell death. There will be some tumor-loaded DCs escaping the restraints from the suppressive cells and cytokines that will enter the lymph nodes as mature DCs capable of mounting a Th1-mediated immunity with warriors such as M1 macrophages, CTLs and natural killer (NK) cells. These cells will migrate to the tumor trying to combat the growing lesion. It has been shown in many cancers that the number of infiltrating lymphocytes positively correlates to survival of the patient [20]. Nevertheless, the lymphocytes are outnumbered by the suppressive cells, and will eventually be anergized or even killed while the M1 macrophages are converted to M2. Hence, the key to a successful immunostimulatory gene therapy is to maintain Th1 immunity. This can be done either by persistent immune activation signals, by blocking resistance mechanisms, or both.

### 2.2. How to Activate and Maintain Th1 Responses

**Figure 2:**
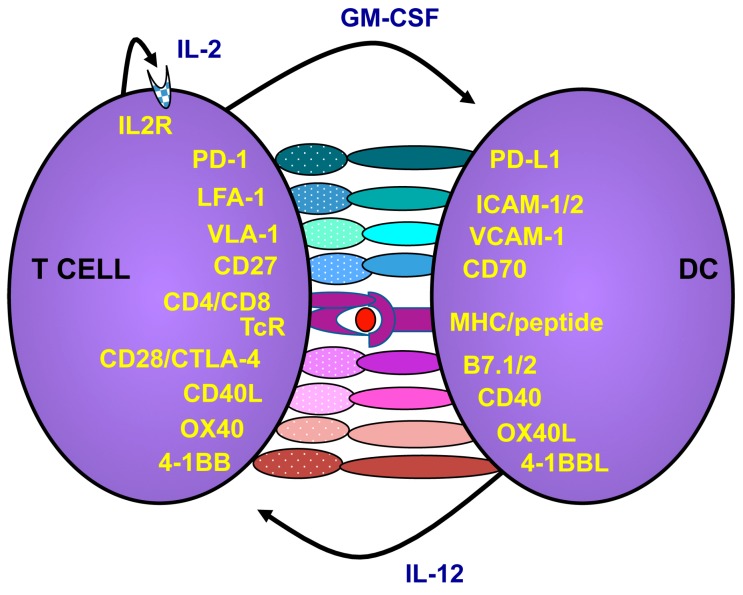
T cells are activated via interactions with antigen presenting cells such as dendritic cells (DCs). When DCs mature they upregulate major histocompatibility complex (MHC) and costimulatory molecules. The T cell recognizing peptides presented by the DC will bind to the DC and receive costimulation. Activated T cells, in turn, express CD40L, which will further stimulate the DCs to increase costimulatory molecules and release cytokines. As an auto-control of immune stimulation, the T cells also express molecules that hamper further activation. For example, PD-1 is upregulated on T cells post activation and if PD-L1 cells are present, the T cells will receive negative signals to restrain activation. Likewise, CTLA-4 will compete with CD28 on binding to the costimulators of the B7 family. However, while CD28 signaling provides costimulation of the T cell, CTLA-4 will block activation. However, the exact mechanism of CTLA-4 is debated.

Th1 responses are mediated via the activation of antigen presenting cells such as DCs. Immature DCs have a high capacity to engulf cell debris and apoptotic cells. Immature DCs can present antigens to T lymphocytes via both major histocompatibility complex (MHC)-I and II, but with low levels of costimulation and no cytokine production the T lymphocytes will be tolerized instead of activated against the presented antigens. In normal, healthy, tissue immature DCs play an important role in maintaining tolerance to our self-tissue and preventing autoimmune reactions. DCs are activated upon danger signaling via different receptors such as Toll-like receptors (TLRs) that recognize pathogen-associated molecular patterns (PAMPs) and receptors that recognize proteins expressed upon danger such as CD40, CD70 and OX40L [21]. For example, adenoviruses can stimulate plasmacytoid DCs via TLR9 to elicit type I interferon (IFN) responses [22]. It is now known that adenoviruses can stimulate multiple TLRs and unknown cytosolic receptors in DCs to elicit robust anti-viral responses [23]. TLR stimulation can be potentiated with stimulation of other receptors such as CD40 [24]. CD40 is stimulated via its ligand (CD40L), which is rapidly expressed in stressed tissues and is one of the most potent DC activators. Upon activation, the DC differentiates into a mature phenotype that is less likely to engulf antigens but instead increases the expression of MHC-I and II costimulatory molecules and begins to produce cytokines including IL12, which promotes Th1 and blocks Th2 induction (Figure 2). DCs interact with CD4+ Th lymphocytes via the binding of MHC-II to the T cell receptor (TcR) and by a number of costimulatory molecules to induce their production of IL2 and CD40L. CD40L on the lymphocytes in turn stimulates the DCs to maintain the Th1 response and further upregulate costimulatory molecules. The fully licensed DC presents antigens to CD8+ T lymphocytes via MHC-I and a wide range of costimulatory molecules and cytokines. These act together with IL2 from Th lymphocytes to increase the cytotoxic capacity and expand the CD8+ T lymphocytes to large clones of antigen-directed CTLs that can seek and destroy their target cells [21].

The immune stimulators first used in immunostimulatory gene therapy were costimulatory molecules targeting DCs (CD40L, B7.1, CD27/CD70), or cytokines to improve lymphocyte function (IL12, IFNγ) both aiming to elicit Th1 immunity [9,10,11,12,13,14]. Molecules that promoted differentiation of myeloid cells such as GM-CSF were also used [25]. Over the years, immunostimulating gene therapies have been evaluated extensively in animal models in which robust Th1-mediated immunity that often was dependent on CD8+ T lymphocytes was induced and the cured mice were protected against subsequent tumor challenge [9,10,11,12,13,14,26,27,28,29,30,31,32]. Several of these approaches led to clinical trials in the late nineties [25,33]. For safety reasons, replication deficient viruses were used as gene vehicles to avoid possible uncontrolled viral infections subsequent to treatment. Still, in the first trials tumor cells were gene engineered *ex vivo* and were used as cellular vaccines. Gene engineered cell vaccines producing GM-CSF, CD40L and IL2 have been used for melanoma, renal cell cancer and various hematological malignancies [25,33,34,35,36,37]. Lately, immunostimulatory gene therapies have been administrated directly to the patients by intravenous infusion, intravesical instillation or intratumoral injection [38,39,40,41,42,43]. Patients with high risk but localized urinary bladder cancer received three bladder instillations of an adenovirus carrying CD40L (AdCD40L) prior to radical cystectomy or resection. A remarkable four of these eight patients had no remaining tumor in the bladder wall, although one of these four had a remaining tumor located in the ureter [41]. However, since the patients with no remaining tumor had radical cystectomy, no long-term results are available. In a study using a murine/human chimeric CD40L (Ad-ISF35), 15 patients with chronic lymphocytic leukemia (CLL) were treated by an intralesional injection. Three patients had partial responses but as many as six patients did not receive any additional therapy for more than six months post virus injection. Most common adverse events in these studies were flu-like symptoms, transient transaminitis and injection site pain [25,33,34,35,36,37,38,39,40,41,42,43]. Many initial trials failed to show efficacy other than transient immune activation, partial responses or stable disease since these studies were performed before the understanding of tumor immune escape mechanisms and the possibility of aiding the immune responses using preconditioning chemotherapy to reduce the levels of these immunosuppressive cells [44]. Further, the responses to immunotherapy follow a different course compared to traditional chemotherapy or irradiation. Initial swelling of the tumor due to inflammation may have been misinterpreted as progression leading to premature interruption of treatment [45]. The combination of preconditioning chemotherapy together with awareness on how to interpret data will likely pave the way for immunostimulatory gene therapy as it has for other immunotherapies.

### 2.3. Targeting the Microenvironment to Promote Cancer Immunotherapy

The next step of immunostimulatory gene therapy is to find targets that tilt the microenvironment to release the restrained natural occurring tumor immunity. As for cancer immunotherapy in general, immunostimulatory gene therapy should be combined with drugs that inhibit MDSCs, Tregs or M2 macrophages. Preconditioning chemotherapy is often given to patients receiving immunotherapy to decrease Tregs and MDSCs [44]. The most commonly used strategy is the combination of cyclophosphamide and fludarabine a few days prior to immunotherapy using T cells [4,5]. Besides the decrease of suppressive cells, chemotherapy-induced lymphocyte or myeloid cell depletion may induce bone marrow cytokine production that restores the immune cell populations (*i.e.*, by homeostatic replication), and this may favor the activation of anti-tumor responses upon immunotherapy. Metronomic cyclophosphamide has been given to patients undergoing immunotherapy in an attempt to control of suppressive immune cells over time [46]. Such supportive chemotherapy protocols may be of great value if they do not hamper the desired anti-tumor responses. Another chemotherapy of interest may be gemcitabine. It is a nucleoside analog that replaces cytidine during DNA replication and leads to growth arrest and apoptosis. Several studies have shown that patients treated with gemcitabine had significantly lower levels of the immunosuppressive molecule TGFβ, Tregs and MDSCs while having a sustained level of activated T cells [47,48]. It has also been shown that gemcitabine potentiated the effect of an oncolytic reovirus [49]. The tyrosine kinase inhibitor (TKI) sunitinib was developed to target signaling in the tumor cells but it was demonstrated that one of the mechanisms of action was a direct inhibitory effect on MDSCs [50,51]. We have previously shown that TKIs such as dasatinib and imatinib also reduce the presence of MDSCs and suppressive factors such as MPO and Arginase I in patients with chronic myeloid leukemia (CML) [52,53]. TKIs are given lifelong to CML patients without serious adverse events in the majority of patients and can be an interesting supporting long-term treatment to immunotherapy. Another small molecule of interest may be lenalidomide. It enhances the degradation of Ikaros 1 and 3, which releases IL2 production [54], and it also inhibits Tregs [55]. There are many attempts to develop more specific drugs that target the microenvironment to release anti-tumor immunity. For example, signal transducer and activator of transcription (STAT)3 is driving MDSCs and M2 macrophages besides driving IL6-mediated tumor cell proliferation. Selective STAT3 inhibitors are being developed [56] and may play an important role in restraining both tumor growth and suppressive myeloid cell populations. Further, molecules that can tilt the M2 macrophages to M1 would be of great interest since they would not only restrain the negative effects of M2 macrophages but would also induce anti-tumor activity by the M1 macrophages. We have previously shown that CD40L gene transfer into the tumor microenvironment tilts the M2 macrophages into M1, at the same time reducing the number of infiltrating MDSCs [57]. CD40 is merging as a hot target in the cancer immunotherapy field since it is able to kick-start many aspects of the immune system to induce tumor immunity [58]. From the field of angiogenesis it has been discovered that a histidine-rich glycoprotein (HRG) inhibits tumor growth and metastasis not only because it regulates tumor vessel abnormalization and has anti-angiogenic properties but because of its capacity to skew M2 macrophages into M1 [59]. This molecule has been inserted into an adenovirus vector and tested as an immunostimulatory gene therapy in experimental settings with promising results [60]. Another molecule in the field of angiogenesis is VEGF. It is overexpressed in the tumor microenvironment and it can be blocked by anti-VEGF antibodies widely used in the clinic for multiple indications [61]. VEGF not only promotes angiogenesis but also prevents infiltration of lymphocytes into the tumor. By blocking VEGF in tumor-bearing mice, CXCL10 and CXCL11 were upregulated followed by a massive infiltration by lymphocytes [62]. Since VEGF can be reduced by sunitinib, this TKI may have multiple actions to improve immunotherapy.

Instead of reducing immunosuppressive cells and their inhibitory molecules, it is possible to make the T lymphocytes resistant to their actions by the use of checkpoint blockade antibodies as described above. A given combination to immunostimulatory gene therapy is the combination of checkpoint blockade antibodies. The concept of combining checkpoint blockade antibodies with immunotherapy has been shown effective in a model of bladder cancer [63]. Interestingly, tumors otherwise resistant to checkpoint blockade antibodies, such as pancreatic cancer, are sensitized by the combination of checkpoint blockade treatment with immunotherapy in a murine pancreatic cancer model [64,65]. There has also been an attempt to combine oncolytic virus therapy with checkpoint blockade by inserting the full-length anti-CTLA4 antibody gene into the virus genome [66]. This may be an interesting approach to localize anti-CTLA4 to the tumor milieu and reduce its systemic toxicity.

### 2.4. Oncolytic Adenoviruses as Immunostimulatory Gene Vehicles

Therapeutic immunostimulatory genes are delivered to the tumor by the use of a great variety of vehicles. Replication deficient adenoviruses have been commonly used since they can carry large transgene cassettes. Unfortunately, transgene expression is of limited duration because adenoviruses do not integrate into the host cell genome. The lack of integration, however, increases safety since the risk for mutagenesis of the host cell is unlikely. Moreover, humans are fully equipped to handle adenoviral infections. For example, most individuals have had upper respiratory tract infections due to adenoviruses and have preformed antibodies against several serotypes, as well as T lymphocytes cross-reactive to all serotypes [67]. For immunostimulatory gene therapy, the immunostimulatory effect of the virus may aid formation of anti-tumor responses by activating TLRs on tumor antigen-loaded DCs [22,23]. Further, cell death by oncolysis is regarded an immunogenic type of death that will provide additional stimulation of DCs via release of damaged-associated molecular pattern (DAMP) such as high-mobility group box 1 (HMGB1) [68]. Upon intratumoral delivery, the virus infects cells in the needle tract and there is a need to increase virus infection to prolong transgene expression. This may be achieved by using oncolytic viruses as gene delivery vehicles. In parallel to the development of immunostimulatory gene therapy a novel field emerged using replicating viruses to combat tumors. The ability of certain viruses to infect cells, propagate and kill them by lysis during the release of new virions means that they can be utilized as cancer therapeutics. To limit oncolysis to tumor cells, the expression of virus replication genes is restricted by adding promoters that are preferentially active in the tumor [8,69,70]. For full benefit, the oncolytic viruses should infect all tumor cells, which can be a challenge if the tumor has metastasized. Since systemic viral spreading to distal tumors may be limited by the immune system, attempts are being made to develop less immunogenic oncolytic viruses. However, instead of decreasing the immunogenicity of oncolytic viruses, another approach is to utilize and boost the intrinsic immunostimulating capacity of the virus by adding immunostimulatory genes into the oncolytic virus genome. Hence, the field of immunostimulating gene therapy and oncolytic virus therapeutics merged. There are different oncolytic viruses that have been armed with immune stimulators, mostly GM-CSF [38,40,71]. For example, a herpes simplex virus armed with GM-CSF (talimogene laherparepvec) has already completed Phase III registration trials in patients with melanoma [71]. Repeated injection of the virus resulted in complete responses in 10.8% of the 295 treated patients, compared to only one complete response in the control arm receiving recombinant GM-CSF. Pre-immunity to herpes simplex virus reduced adverse reactions such as flu-like symptoms showing that immediate responses to the virus vehicle are occurring [71,72]. However, since the virus is locally administered pre-immunity does not seem to interfere with virus infection and efficacy. The role of GM-CSF in cancer immunotherapy has been debated since it expands MDSCs. GM-CSF-producing or treated tumors are infiltrated with MDSCs rather than DCs (as reviewed in [73]). However, the combination of GM-CSF with the activating signaling from the virus may tilt the myeloid cells in the right direction of inducing tumor immunity. Indeed, talimogene laherparepvec induced immune activation in parallel to reduced level of MDSCs and Tregs in patients [74]. The next step of talimogene laherparepvec treatment is the combination with checkpoint blockade antibodies and there is initial data in humans using such a combination [75].

There are advances using oncolytic adenoviruses as gene delivery vehicles since they are highly immunogenic and their immunogenicity will further boost anti-tumor responses. However, the full efficacy and toxicity of the combined oncolysis and immune stimulation is difficult to determine in murine models since oncolysis is limited in murine cells and the immunostimulatory effect cannot be evaluated in xenograft models using human tumor cells in immunodeficient mice. However, oncolytic adenoviruses expressing either GM-CSF or CD40L have reached clinical evaluation and demonstrated both feasibility and safety upon intratumoral or systemic delivery [38,39,40,42]. The choice of an immunostimulatory gene vehicle such as adenoviruses will limit systemic spread of the virus due to preformed antibodies in the patients. Even if the virus could be efficiently administrated by intravenous infusion the first time, antibodies will rapidly be formed to prevent efficient spreading of the virus for the upcoming treatments. However, we have noted less antibody formation when the virus is given locally in the tumor microenvironment [41] and since the main effect of the treatment will be immune activation and not oncolysis *per se*, the net outcome will still be systemic since the immune cells will patrol the whole body to seek and destroy tumor cells. Even if a less immunogenic virus would be chosen for delivery of an immunostimulatory gene, subsequent immunity against the virus will likely be developed due to the immunostimulatory gene. Hence, it may be better to aim for as much immunity boost as possible by combining a highly immunogenic virus with a great Th1 stimulatory gene to elicit long lasting anti-tumor immunity.

## 3. Concluding Remarks

Cancer immunotherapy is in the limelight and is predicted as the next cornerstone of cancer therapeutics. CAR T cells and checkpoint blockade antibodies have shown remarkable long lasting complete responses in patients but there are still patients not responding to treatment or that relapse rapidly after treatment. After decades of mapping the tumor microenvironment, developing novel immunostimulatory strategies and finding means to hamper immunosuppressive cells we now have a giant tool box for evaluating combination therapeutics to determine the best options to boost the immune system. Immunostimulating gene therapy using oncolytic adenoviruses as vehicles is an appealing choice due to the combined efforts of the adenovirus backbone and the immunostimulatory transgenes to induce DC activation and the subsequent activation of cytotoxic lymphocytes together with the oncolytic capacity of the virus.
